# Steam-based thermotherapy for managing nematodes in strawberry transplants

**DOI:** 10.21307/jofnem-2020-095

**Published:** 2020-11-06

**Authors:** Churamani Khanal, Mengyi Gu, Natalia A. Peres, Johan A. Desaeger

**Affiliations:** 1 Department of Plant and Environmental Sciences, Clemson University, Clemson, South Carolina, 29634; 2 Entomology and Nematology Department, Gulf Coast Research and Education Center, University of Florida, Wimauma, Florida, 33598; 3 Plant Pathology Department, Gulf Coast Research and Education Center, University of Florida, Wimauma, Florida, 33598

**Keywords:** Nematodes, Non-chemical, Management, Strawberry, Thermotherapy

## Abstract

Aerated steam-based thermotherapy was developed and evaluated for its efficacy in managing three nematode species (*Aphelenchoides besseyi*, *Meloidogyne hapla*, and *Pratylenchus penetrans*) that are often transported as quiescent passengers on strawberry transplants shipped to Florida from out-of-state nurseries. Initial studies were focused on evaluating the intrinsic temperature sensitivity of each nematode species to hot water in laboratory conditions. Each nematode species was exposed to hot water at 40, 44, 48, and 52°C for 1, 5, 10, 30, 60, 120, and 240 min. Exposure for 60 min or higher at 40°C paralyzed all three nematode species when examined immediately after heat treatment. Examination of the nematodes 24 hr post-treatment suggested that 100% mortality of all three nematode species was achieved when nematodes were exposed to hot water at a minimum temperature of 44°C for 120 min. Further studies were conducted to evaluate the efficacy of aerated steam to kill all three nematode species by exposing nematode-infested strawberry transplants at 44°C for 60, 120, and 240 min. Exposure of nematode inoculated plants to steam for 60 or 120 min reduced the populations of all three nematode species, but this was not enough to completely eradicate any of the three nematode species. Exposure for 240 min, however, was the most effective in reducing the populations of the three nematode species. A 240 min of exposure to aerated steam completely eradicated *A. besseyi* and *M. hapla* while *P. penetrans* populations were reduced only by 85%. Furthermore, the aerated steam had minimal to no adverse effect on plant biomass. Results from both the laboratory and greenhouse studies indicated that *M. hapla* was more sensitive to heat treatment followed by *A. besseyi* and *P. penetrans*. Results from this study suggested that aerated steam-based thermotherapy has good potential as a non-chemical method of management of nematodes of strawberry transplants.

Strawberry is one of the major fruit crops in the United States with a farm gate value of approximately $2.7 billion (USDA NASS, 2019). Florida represents approximately 20% of the total strawberry planting acreage (USDA NASS, 2019) making it the second largest producer of strawberry in the United States. Additionally, Florida is the most important producer of strawberry during winter due to its subtropical climate, and this state produces virtually all strawberries available in winter months.

Each year more than 100 million strawberry transplants are shipped to Florida predominantly from California, North Carolina, and Canada to be planted in the winter strawberry production fields. Strawberry transplants coming into Florida from out-of-state nurseries often harbor many pathogens including nematodes (Noling and Whidden, 2010; Nyoike et al., 2012; Oliveira et al., 2017; Forcelini and Peres, 2018). The nematodes associated with the shipped strawberry transplants are foliar nematode (*Aphelenchoides besseyi* Christie, 1942), root-knot nematode (*Meloidogyne hapla* Chitwood, 1949), and root-lesion nematode (*Pratylenchus penetrans* Cobb, 1917). These nematodes coming as quiescent passengers on strawberry transplants do not typically cause any obvious damage symptoms, however, nematode damage occurs later in the crop growing season when environmental conditions are favorable for nematode reproduction. In 2016, foliar nematode outbreak occurred in some strawberry farms in Florida and caused significant crop damage (Desaeger and Noling, 2017). The temperate nematodes, *M. hapla* and *P. penetrans*, are now also increasingly found in Florida strawberry farms. Both nematodes are not common in Florida, and were likely introduced to the state with infested planting material from the north.

Currently available nematode management methods for strawberry production are mostly limited to the use of soil fumigants, and more recently also some newly registered non-fumigant nematicides (Watson and Desaeger, 2019). Because of the high nematode pressure in Florida strawberry fields, especially of sting nematode (*Belonolaimus longicaudatus* Rau), strawberry production is heavily reliant on the use of chemical nematicides. Host plant resistance for nematodes is not available in strawberry, and other management options such as biologicals, crop rotation, and cover cropping may not always be adequate or practical. Certainly, there are many health, environmental, and economic concerns about soil fumigation, and the practice is becoming increasingly scrutinized. Moreover, while soil fumigation may provide good control of resident nematode populations, such as sting nematodes, this may not be the case for nematodes and other pathogens that are being introduced with transplants. The biological vacuum created by soil fumigation may actually provide a perfect habitat for such introduced nematodes to proliferate. Therefore, an effective and sustainable nematode management program must start with the employment of nematode free planting materials. A non-chemical and environmentally friendly alternative for the management of nematodes of strawberry transplants that may be implemented prior to planting is thermotherapy. Conventionally, thermotherapy is carried out using hot water. This hot water-based thermotherapy has been practiced since the mid-1930s for the management of diseases associated with planting materials, particularly viral diseases (Kunkel, 1935). This approach is still employed to manage diseases caused by viruses, bacteria, fungi, or nematodes in several fruit crops (Burr et al., 1996; Tsang et al., 2001; Turechek and Peres, 2009). Although hot water treatment is commonly employed to manage pathogens, it has some limitations. Hot water treatment is often associated with an adverse effect on the agronomic performance of the crop (Buchner, 1991). Additionally, there is a high risk of cross-contamination of pathogens or insects from diseased to healthy plants during hot water treatment. An improved thermotherapy method that employs aerated hot steam instead of hot water has the potential to overcome the drawbacks associated with hot water treatment (Ghatrehsamani et al., 2019; Wang et al., 2019). The main objective of this study is to investigate the potential of aerated hot steam-based thermotherapy to manage *A. besseyi*, *M. hapla*, and *P. penetrans* on strawberry transplants.

## Materials and methods

### Laboratory assay

Laboratory assays were conducted to determine the effect of hot water treatment on mortality of *A. besseyi*, *P. penetrans*, and *M. hapla*. All nematodes were originally isolated from strawberry fields near Plant City, Florida (during the past 2-4 years). Pure cultures of these nematode species have been maintained on suitable hosts in greenhouse and growth rooms. In total, 20 vermiform life stages of each nematode species were handpicked and placed separately in 10 ml water in 20 ml glass vials. The treatments for this experiment included three nematode species, four temperature levels (40, 44, 48, and 52°C) and seven exposure times (1, 5, 10, 30, 60, 120, and 240 min). At each temperature, the glass vials were placed in a constant temperature water bath (Blue M Electric Company, Illinois, USA). Nematode mortality was evaluated immediately following exposure, as well as 24 hr post thermotherapy. A nematode was considered paralyzed when no movement was observed immediately after heat treatment. Similarly, a nematode was considered dead when no movement could be observed upon teasing with a needle at 24 hr following the heat treatment. Each treatment was replicated three times and the entire experiment was repeated once.

### Greenhouse assay

Strawberry transplants (cultivar Sensation^TM^ Florida127) obtained from a commercial nursery in California were planted in 11 cm wide × 13 cm tall plastic pots in a greenhouse. Each plastic pot received 1 kg of soil (95% sand, 2% silt, 3% clay with 0.9% organic matter, A & L Western Laboratories, Inc., CA) sterilized at 185°C for 2 hr. The pots were kept under a mist chamber for two weeks to speed the crop establishment process prior to inoculation with nematodes. Misting was programmed to mist every 10 min for 6 sec (K RAIN, Florida, USA). The inoculum level used was, respectively, water suspension of 500, 300, and 125 vermiform life stages of *A. besseyi*, *P. penetrans*, and *M. hapla* per plant. *Aphelenchoides besseyi* was inoculated on the foliage while *P. penetrans* and *M. hapla* were pipetted into three depressions arranged into a triangular pattern surrounding the plant. Strawberry transplants inoculated with *A. besseyi* and *M. hapla* were steam-treated a week after inoculation, while the ones inoculated with *P. penetrans* were steam-treated a month after inoculation. For steam treatment, strawberry roots were separated from the soil, gently washed with water, and the plants were placed on autoclave standard plastic trays in a steam chamber that generated aerated steam. The treatment process included a pre-treatment phase of 37°C for 60 min (Brown et al., 2016), followed by 60 min cool down at the then ambient temperature, and subsequent heat treatment at 44°C for 60, 120, or 240 min (Wang et al., 2019). The steam-treated plants were replanted in new plastic pots containing 1 kg of sterilized soil in the greenhouse with ambient temperatures of 33 ± 5°C. Experiments were conducted during September 2019 to February 2020 under natural light and humidity conditions in the greenhouse (no artificial light or humidity control). Standard irrigation and fertilization were conducted. The experiment was established as a completely randomized design with five replications. The entire experiment was repeated once.

The experiment was terminated 60 days after steam treatment. Nematodes were extracted from a 200 cc subsample of soil from each pot using the salad spinner method as described by Khanal and Desaeger (2020). For the experiments involving *A. besseyi*, foliage from each plant was chopped into 2.5 cm pieces, soaked in water in a 500 ml glass beaker for a week, and nematodes were collected by passing the solution through 25 µm sieve. Extraction of *P. penetrans* from strawberry roots was conducted by chopping the roots into 2.5 cm pieces, placing the roots in water in 500 ml conical flasks supplied with constant air flow, and collecting the nematodes every day for a week. Extraction of *M. hapla* eggs from strawberry roots was conducted by agitating the entire root system in 0.6% NaOCl for 4 min (Hussey and Barker, 1973). All the extracted nematodes and eggs were enumerated using an inverted microscope (Axio Vert.A1, Zeiss, Jena, Germany) at 50 x magnification. Plant biomass was dried at 55°C for a week and weighed.

### Data analysis

Data obtained from laboratory and greenhouse assays were analyzed using mixed model in JMP PRO 14.3 (SAS Institute, Cary, NC) as factorial analysis (nematode species X exposure time). Nematode species, exposure time, nematode reproduction, and plant dry weight were used as fixed effects, and replication was used as a random effect. Tukey’s HSD test (*P* ≤ 0.05) was used as post hoc mean comparisons. When there was significant interactive effects of nematode species and exposure time on nematode activity (percentage paralysis or mortality) at specific temperatures, regression analysis was conducted using fit *Y* by *X* module to determine the specific effect of each factors on nematode activity.

## Results

### Laboratory assay

The immediate effect of hot water on the activity of nematodes expressed as a percentage of paralyzed nematodes is presented in Table 1. None of the nematode species were 100% paralyzed when exposed at 40°C for any exposure time. Across all exposure times, 100% of root-knot nematodes were paralyzed at or above 44°C, while foliar nematodes required 52°C to provide the same result. Exposure time significantly affected nematode activities at 48°C, and the percentage of paralyzed nematodes across all nematode species at this temperature ranged from 96.9 to 100%. Across all species, an exposure of 10 min or higher at 48°C was enough to paralyze 100% of the nematodes. At 52°C, 100% of foliar and root-knot nematodes and 99.9% of root-lesion nematodes were paralyzed. Across all nematode species, nematodes were completely paralyzed when exposed for 60 min or longer at 44°C or above.

**Table 1.** Effect of hot water on paralyzed nematode percentage of three nematode species (*Aphelenchoides besseyi*, *Pratylenchus penetrans*, and *Meloidogyne hapla*; *n* = 20) immediately after treatment at 40, 44, 48, and 52°C for 1, 5, 10, 30, 60, 120, and 240 min.

Factor	Treatment level	40°C	44°C	48°C	52°C
Nematode (N)	*A. besseyi*	50.8b	70.0c	99.3a	100.0a
	*P. penetrans*	47.1b	75.7b	98.9a	99.9a
	*M. hapla*	63.0a	100.0a	100.0a	100.0a
Exposure time (T)	1 min	1.1e	45.3d	96.9b	99.7a
	5 min	10.8d	56.4c	98.9ab	100.0a
	10 min	33.1c	77.2b	100.0a	100.0a
	30 min	58.6b	94.4a	100.0a	100.0a
	60 min	88.9a	100.0a	100.0a	100.0a
	120 min	90.8a	100.0a	100.0a	100.0a
	240 min	92.2a	100.0a	100.0a	100.0a
*P* value	N	< 0.0001	< 0.0001	0.1075	0.3714
	T	< 0.0001	< 0.0001	0.0005	0.4295
	N × T	< 0.0001	< 0.0001	0.2769	0.4545

**Notes:** Data were combined over two experiments and are means of six replications. Data were analyzed with ANOVA and Tukey’s HSD (*P* ≤ 0.05). Within columns, means followed by a common letter are not significantly different.

**Figure 1:**
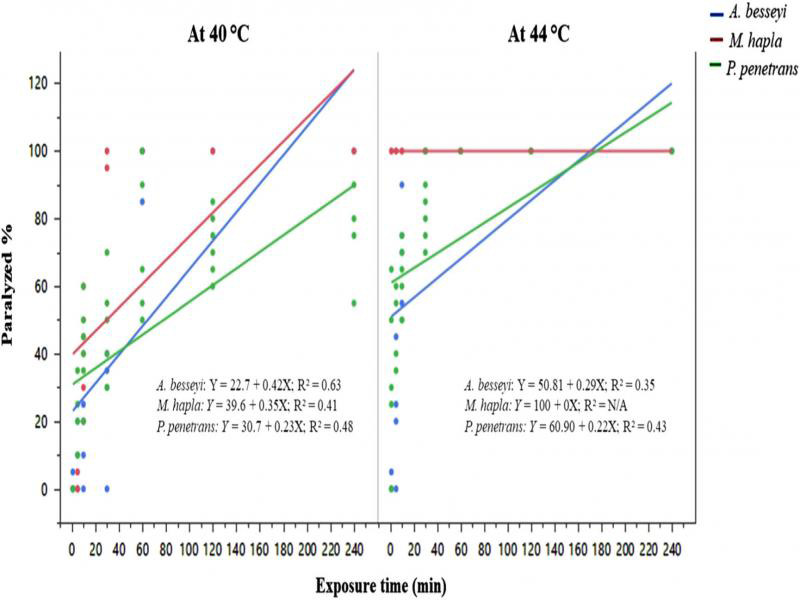
Paralyzed nematode percentage of three nematode species (*Aphelenchoides besseyi*, *Pratylenchus penetrans* and *Meloidogyne hapla*; *n* = 20) immediately after heat treatment at temperatures 40 and 44°C for various exposure times. Data were combined over two experiments and are means of six replications.

The percentage of paralyzed nematodes was influenced by interactive effects of nematode species and exposure time, which is presented in Figure 1. At 40°C, 100% of the root-knot and foliar nematodes, but only 76.7% of root-lesion nematodes, were paralyzed when exposed for 240 min. At 44°C, all root-knot, foliar, and root-lesion nematodes were paralyzed when exposed for 60 min or longer.

The effect of hot water on mortality of nematodes 24 hr post thermotherapy is presented in Table 2. There were significant main and interactive effects of nematode species and exposure time on mortality of nematodes at 40°C, 44°C, and 48°C. Across all nematode species, the greatest mortality obtained at 40°C was 95% when exposed for the longest time (240 min). Mortality of nematodes at 48°C across all species ranged from 4.7 to 100%, with the exposure for 120 min or higher providing 100% mortality. The exposure of nematodes at 44°C, across all species, provided 89.7% mortality for 30 min treatment and 98.9% mortality for 60 min treatment, and the effect was not significantly different from the exposure at 120 and 240 min. At 48°C and across all exposure times, mortality of 100, 99.5, and 98.8% was achieved for root-knot, foliar, and root-lesion nematodes, respectively. Treatment at 48°C for 10 min or higher, across all species, resulted in 100% mortality of nematodes. Any significant main and interactive effects of nematode species and exposure time were not observed as 99.9% of root-lesion nematode and 100% of foliar and root-knot nematodes were killed at this temperature. Across all nematode species, 100% mortality was obtained when exposed for 5 min or higher at 52°C.

**Table 2.** Effect of hot water on mortality percentage of three nematode species (*Aphelenchoides besseyi*, *Pratylenchus penetrans*, and *Meloidogyne hapla*; *n* = 20) 24 hr post heat treatment at 40, 44, 48, and 52°C for 1, 5, 10, 30, 60, 120, and 240 min.

Factor	Treatment level	40°C	44°C	48°C	52°C
Nematode (N)	*A. besseyi*	45.4a	68.9b	99.5ab	100.0a
	*P. penetrans*	23.5c	62.4c	98.8b	99.9a
	*M. hapla*	31.1b	86.9a	100.0a	100.0a
Exposure time (T)	1 min	1.7e	4.7d	96.7b	99.7a
	5 min	4.2e	40.6c	99.4a	100.0a
	10 min	6.1de	75.3b	100.0a	100.0a
	30 min	17.8cd	89.7a	100.0a	100.0a
	60 min	28.9c	98.9a	100.0a	100.0a
	120 min	79.4b	100.0a	100.0a	100.0a
	240 min	95.0a	100.0a	100.0a	100.0a
*P* value	N	< 0.0001	< 0.0001	0.0007	0.3714
	T	< 0.0001	< 0.0001	< 0.0001	0.4294
	N × T	< 0.0001	< 0.0001	< 0.0001	0.4543

**Notes:** Data were combined over two experiments and are means of six replications. Data were analyzed with ANOVA and Tukey’s HSD (*P* ≤ 0.05). Within columns, means followed by a common letter are not significantly different.

**Figure 2:**
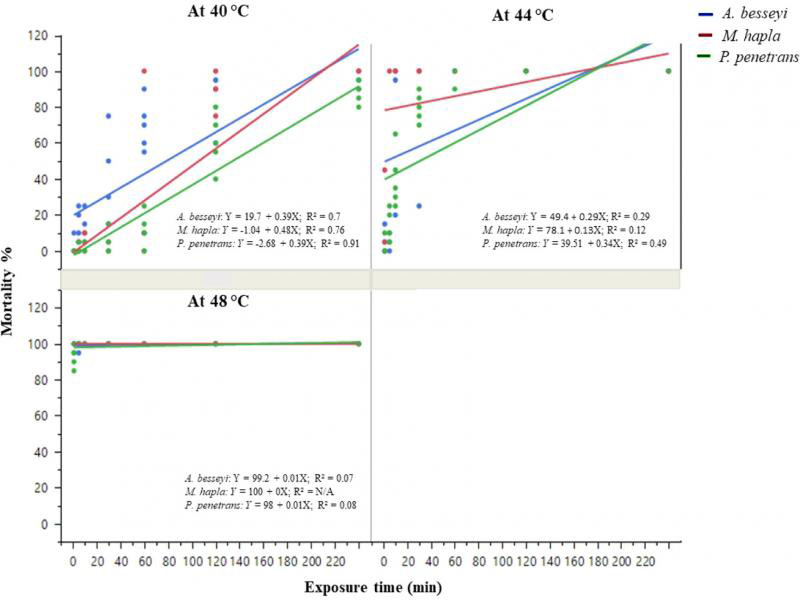
Mortality percentage of three nematode species (*Aphelenchoides besseyi*, *Pratylenchus penetrans* and *Meloidogyne hapla*; *n* = 20) 24 hr after heat treatment at temperatures 40, 44, and 48°C for various exposure times. Data were combined over two experiments and are means of six replications.

The mortality of nematodes was influenced by interactive effects of nematode species and exposure time, which is presented in Fig 2. At 40°C, only the longest exposure (240 min) provided 100% mortality of root-knot nematode, while 88.3 and 96.7% mortality was achieved for root-lesion and foliar nematodes, respectively. The minimum exposure times required to achieve 100% mortality of root-knot, foliar, and root-lesion nematode at 44°C were, respectively, 5, 60, and 120 min. A 1-min exposure for root-knot nematode, 5 min exposure for root-lesion nematode and 10 min exposure for foliar nematodes was enough to achieve 100% mortality at 48°C.

### Greenhouse assay

The effect of aerated steam on population of *A. bessseyi* and foliage dry weight of strawberry is presented in Table 3. The population of *A. besseyi* ranged from 0 to 1267 vermiform life stages per pot. Exposure of *A. besseyi* inoculated plants to aerated steam for 60 min did not significantly reduce the nematode population in relation to uninoculated control, however, exposure for 120 min significantly reduced the nematode population. A 240 min exposure completely killed the inoculated nematodes. There was no significant effect of heat treatment on foliage dry weight which ranged from 1.8 to 2.9 g.

**Table 3.** Effect of aerated steam at 44°C on reproduction of three nematode species (*Aphelenchoides besseyi, Pratylenchus penetrans* and *Meloidogyne hapla)* and plant dry weight (g) of strawberry under various exposure times^a^.

	A. besseyi		P. penetrans		M. hapla		
Treatment	Nematodes per pot	Foliage dry wt.	Nematodes per pot	Plant dry wt.	Nematodes per pot	Eggs per root system	Plant dry wt.
60 min exposure	953a	2.2a	225b	3.7ab	3b	480b	5.1a
120 min exposure	78b	1.8a	145bc	3.8ab	0.1b	552b	4.8a
240 min exposure	0b	2.4a	69bc	3.1b	0b	0b	5.5a
Inoculated untreated control	1267a	1.8a	462a	4.5a	67a	19042a	6.1a
Uninoculated untreated control	0b	2.9a	0c	4.9a	0b	0b	5.0a
*P* value	< 0.0001	0.1163	< 0.0001	0.0032	< 0.0001	< 0.0001	0.7167

**Notes:** Data were analyzed with ANOVA and Tukey’s HSD (*P* ≤ 0.05). Within columns, means followed by a common letter are not significantly different. ^a^The inoculum constituted, respectively, 500, 300, and 125 vermiform life stages of *A. besseyi*, *P. penetrans* and *M. hapla* per plant.

The application of aerated steam had a significant effect on reproduction of *P. penetrans* and *M. hapla* as presented in Table 3. The reproduction of *P. penetrans* ranged from 69 to 462 per pot. Exposure of *P. penetrans* inoculated strawberry to aerated steam for 60, 120, and 240 min significantly reduced the nematode reproduction compared to the inoculated untreated control, the reduction being 51, 69, and 85%, respectively. A significant effect of aerated steam was also observed on reproduction of *M. hapla*. The reproduction of *M. hapla* ranged from 0 to 67 vermiform life stages per pot, which is very low compared to that of *A. besseyi* and *P. penetrans*. The number of *M. hapla* eggs was also significantly affected by the steam treatment, the reproduction ranging from 0 to 19,042 per root system. Exposure of *M. hapla* inoculated strawberry plants for 60 and 120 min significantly reduced the nematode reproduction (vermiform life stages and eggs) compared to the inoculated untreated control, while a 240 min exposure completely killed the nematode.

The effect of aerated steam on plant dry weight of *P. penetrans* and *M. hapla* inoculated strawberry is presented in Table 3. There was a significant effect of aerated steam on plant dry weight of *P. penetrans* inoculated strawberry plants, however, no effect was observed on *M. hapla* inoculated plants. A significant reduction in plant dry weight of *P. penetrans* inoculated plants treated for 240 min was observed when compared to that of inoculated untreated control, the reduction being 31%. Exposure of *P. penetrans* inoculated plants for 60 or 120 min did not significantly reduce the plant dry weight.

## Discussion

Exposure of nematode-inoculated strawberry transplants to aerated steam for 240 min at 44°C was the most effective, and completely eradicated *A. besseyi* and *M. hapla* but not *P. penetrans*. Results from inoculated plants confirmed results from our hot water treatments, where *P. penetrans* also showed to be more tolerant of higher temperatures. It also should be pointed out that, due to an issue with the operation of the steam chamber, the plants inoculated with *P. penetrans* were steam-treated three weeks later than those inoculated with the other two nematode species. This could possibly have affected the efficacy of the heat treatment. Any previous studies on the efficacy of steam treatment for managing nematodes are currently not available, except for a modified heat treatment method known as controlled atmosphere temperature treatment (CATT) that employs heat treatment in high humid conditions and is practiced in the Netherlands to manage nematodes and mites of strawberry planting stocks (van Kruistum et al., 2012).

Temperature plays a critical role in the growth and development of nematodes. The order of tolerance to cold in root-knot nematodes has been reported as *M. chitwoodi* > *M. hapla* > *M. incognita* > *M. arenaria* > *M. javanica* (Van, 1985; Dávila-Negrón and Dickson, 2013). East et al. (2019) found that *M. hapla* reproduction was maximum in early March when average soil temperature in Washington is 7°C. However, it is likely that *M. hapla* populations in Florida have different temperature developmental dynamics than those in more northern regions as this nematode damages Florida strawberries during February to March when average soil temperature is 25°C. Daulton and Nusbaum (1961) determined that *M. hapla* eggs in dry soil were rapidly killed at temperatures 36 and 40°C. Similarly, Khanal and Desaeger (2020) stated that high soil temperature during summer can greatly suppress some of the more cryophilic nematodes in Florida, such as *M. hapla*. Results from our study indicated that a 120 min or greater exposure of nematodes to heat at 44°C or higher will kill these nematodes. *Meloidogyne hapla* is quite common in Florida strawberry fields, and typically shows up toward the end of the season, around February to March (Desaeger, 2018). Although the nematode can cause significant damage to strawberry, its economic impact is probably less than for sting nematodes, which attack the crop earlier in the season, when plants are younger and more susceptible. *Meloidogyne hapla*, however, can cause severe damage to relay- or double-cropped cucurbits (cantaloupe, squash, cucumber), which are commonly planted in strawberry beds around March to April (Khanal and Desaeger, 2020). Very poor crop establishment has been observed when these double crop are direct-seeded in strawberry beds that have high populations of *M. hapla* (Desaeger, 2018). As the soil temperature in spring rapidly increases in Florida, it slows down the reproductive capacity of northern nematodes such as *M. hapla*, and soil population build-up and crop damage typically decreases after and when the double-crop can be properly established (Khanal and Desaeger, 2020). Although *M. hapla*, and to a lesser extent *P. penetrans*, are already present in several strawberry fields in Florida, strawberry growers would still benefit from thermotherapy by reducing further spread and inoculum build-up of these nematodes.

The aerated steam-based thermotherapy technique is an improved method of conventional thermotherapy which involves hot water. The hot water-based thermotherapy is a century-old method and is still being employed to eradicate pathogens of planting materials including nematodes (Kunkel, 1935; Qiu et al., 1993; Burr et al., 1996; Brcka et al., 2000; Tsang et al. 2001; Turechek and Peres, 2009). Although the conventional hot water-based thermotherapy method has been commonly practiced on other crops such as citrus and ornamental plants, its use on strawberry is limited. The hot water treatment is often associated with an adverse effect on the crop (Buchner, 1991) and the treatment process can lead to cross-contamination of pathogens from diseased to healthy plants. Results from our study suggest that steam-based thermotherapy is a relatively safe method with very little to no adverse effect on strawberry plants. Our results are in agreement with the findings of Wang et al. (2019) who reported that steam-based thermotherapy conducted at 44°C for 240 min did not adversely affect the agronomic characters of strawberry plants. Wang et al. (2019) also found that steam-treated transplants had better crop establishment and flowered earlier in relation to the untreated plants, suggesting that thermotherapy can actually have beneficial effects on plants. Based on the previous findings as well as the findings from our experiments involving *A. besseyi* and *M. hapla*, it would probably be reasonable to argue that adverse effects of steam treatment (44°C for 240 min) on *P. penetrans*-inoculated strawberry transplants was likely caused by the unexpected delayed steam treatment which extended the crop establishment period by three weeks. This may indicate that older transplants are less tolerant of heat treatment, or that *P. penetrans* somehow makes the plants more susceptible to heat stress. Further studies are needed to confirm this.

Thermotherapy, also known as ‘passive heating’ in medical science, has been proven to be beneficial in curing metabolic diseases in humans as the gradual heating process activates heat shock proteins, a protein family that is associated with the adaptation to heat stress (Faulkner et al., 2017). The pre-treatment phase of thermotherapy in plants is similar to the passive heating in humans, and the process has been shown to activate heat shock proteins in the plants as well, thereby enabling plants to withstand gradual heat stress (Gulen and Eris, 2015; Brown et al., 2016). A precise temperature control during the therapeutic phase facilitates the use of higher temperatures with less risk of injury to the plants. While precise heat control activates the heat shock proteins in plants, the question remains whether the same therapeutic temperatures would simultaneously activate the heat shock proteins in nematodes thereby increasing the ability of nematodes to endure higher temperatures. Reduced mortality (85%) of *P. penetrans* in our study could have possibly been associated with activation of heat shock proteins in this nematode as the same treatment completely killed *A. besseyi* and *M. hapla.* However, further study is needed to validate this phenomenon in nematodes.

The adverse effects of chemical nematicides, especially soil fumigants, on the environment and human health, their increasing cost and strict application guidelines have made them less desirable for growers (Khanal et al., 2018; Khanal and Desaeger, 2020). Other non-chemical methods, such as host plant resistance are currently not available in Florida strawberry cultivars. The aerated steam-based thermotherapy has great potential to become part of an integrated nematode management program for strawberry. This method serves as a first line of defense by managing potentially damaging nematodes at the pre-planting stage, and reducing the risk of introducing them in strawberry production fields. It would also reduce the need for post-plant nematicide applications. In addition, steam-based thermotherapy has also proven to be effective for the management of other pathogens and insects that are present in the host or carried along with the planting materials (van Kruistum et al., 2012; Zuniga and Peres, 2017; Ghatrehsamani et al., 2019; Wang et al., 2019; Renkema et al., 2020).

Results from our study suggested that the aerated steam-based thermotherapy can be very effective in reducing or eradicating nematodes inside strawberry transplants, and reduce the introduction of these nematodes into strawberry production fields. More research is needed to evaluate the technique on a commercial scale and efforts are underway to evaluate this. Several commercial strawberry nursery growers are currently evaluating the technology in their fields, and we expect that in the near future strawberry growers in Florida production fields will follow suit.
